# Optimised metrics for CRISPR-KO screens with second-generation gRNA libraries

**DOI:** 10.1038/s41598-017-07827-z

**Published:** 2017-08-07

**Authors:** Swee Hoe Ong, Yilong Li, Hiroko Koike-Yusa, Kosuke Yusa

**Affiliations:** Wellcome Trust Sanger Institute, Hinxton, Cambridge CB10 1SA UK

## Abstract

Genome-wide CRISPR-based knockout (CRISPR-KO) screening is an emerging technique which enables systematic genetic analysis of a cellular or molecular phenotype in question. Continuous improvements, such as modifications to the guide RNA (gRNA) scaffold and the development of gRNA on-target prediction algorithms, have since been made to increase their screening performance. We compared the performance of three available second-generation human genome-wide CRISPR-KO libraries that included at least one of the improvements, and examined the effect of gRNA scaffold, number of gRNAs per gene and number of replicates on screen performance. We identified duplicated screens using a library with 6 gRNAs per gene as providing the best trade-off. Despite the improvements, we found that each improved library still has library-specific false negatives and, for the first time, estimated the false negative rates of CRISPR-KO screens, which are between 10% and 20%. Our newly-defined optimal screening parameters would be helpful in designing screens and constructing bespoke gRNA libraries.

## Introduction

The discovery of the bacterial adaptive immune system, namely clustered regularly interspaced short palindromic repeats (CRISPR)-Cas9 system^1–5^, and its subsequent development^6–9^ into a genome editing tool have made possible the precise targeting and modification of DNA, including in mammalian cells. In addition to genome editing, the high versatility of the CRISPR-Cas system has led to the development of various derivative tools for epigenome^10–12^ and base editing^13, 14^, as well as transcriptional activation/silencing^15–18^.

We and others have exploited the highly consistent editing efficiency of the CRISPR-Cas system and the scalable nature of gRNA vector production, and developed genome-wide CRISPR-knockout (KO) screening^19–21^. In CRISPR-KO screening, a pool of synthetic single guide RNAs (sgRNAs, or simply gRNAs) that targets every gene in the genome under interrogation is transduced into the cells under study, usually at the stoichiometry of a single gRNA per cell. The final cell population that exhibits the phenotype of interest are then recovered and the integrated gRNAs, which reveal the identity of the knocked-out genes, are sequenced in parallel. Analysis of the differences in the abundance of gRNAs between the controls and the treated cells would unveil the relationship between genes and the phenotype of interest^22^.

Several factors need to be considered when constructing CRISPR gRNA libraries for effective genome-wide CRISPR-KO screening. First and foremost is the selection of gRNAs to be included in a library, as pooled screening requires the gRNAs to be effective in knocking out only the target genes without affecting other loci which may share high sequence similarity. Various algorithms for selecting suitable gRNAs with minimal off-target activity have been reported^23–27^. In addition to off-target effects, it is now evident that different gRNAs exhibit varying KO efficiency due to different on-target cutting efficiency^20, 28–31^ and/or DNA repair outcome^32^. Lastly, genetic variations present in individuals or cell lines may also affect on-target activity. It is therefore important to choose gRNAs that target common sequences within the human population and to ensure that every gene has a fair chance of being knocked out in various human cell lines.

In this study, we seek to examine the vital design parameters that affect screening efficiency. We first compared the performance of three second-generation human genome-wide CRISPR libraries that employed different gRNA selection strategies, and used the results to arrive at an estimate of the false negative rate of CRISPR-KO screens. We then proceeded to determine the optimum number of gRNA per gene and the number of replicates that provide the best trade-off for genome-wide CRISPR-KO screens.

## Results

### Screening performance of second-generation CRISPR libraries

**Figure 1:**
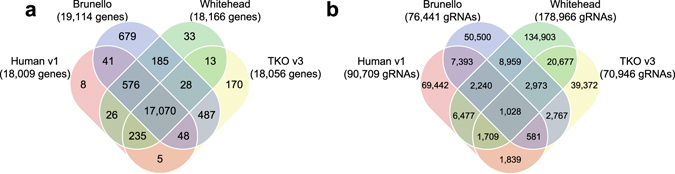
Overlaps of targeted human genes and 19-mer gRNAs of second-generation CRISPR libraries. (**a**) Venn diagram showing overlaps of human genes targeted by four second-generation genome-wide CRISPR libraries. (**b**) Venn diagram showing overlaps of 19-mer gRNAs in four second-generation genome-wide CRISPR libraries. gRNA sequences of the 3′ 19-mers were used in order to make the 19-mer gRNAs in the Human v1 library comparable to the 20-mer gRNAs in the other three libraries.

To date, 7 genome-wide CRISPR gRNA libraries targeting human genes have been described^27, 31, 33–37^. Of these, 4 libraries were designed and constructed with features that improve gRNA efficacy, thereby increasing screen performance^27, 31, 36, 37^. In this study, we refer to these 4 libraries as second-generation CRISPR libraries (Table 1 and Fig. 1). Apart from differences in the constituent gRNAs and a small variation in the human genes targeted (Fig. 1), two other major differences among these libraries are (1) the use of on-target prediction for gRNA selection^20, 27^ and (2) the gRNA scaffold^38^ used. The number of gRNAs targeting each gene also differs among the 4 libraries (Table 1). We directly compared the performance of 3 second-generation libraries in an identical condition to examine the effect of these major differences on screen outcomes.

**Table 1:** List of second-generation human genome-wide CRISPR libraries.

Library	Design principle	Scaffold	gRNAs*	Genes	gRNAs per gene	gRNA length (bases)	Addgene ID	Reference
Human v1	Avoidance of T stretches; no on-target prediction	Improved	90,709	18,009	~5	19	67989	35
Brunello	On-target prediction (Rule set 2) and off-target prediction	Conventional	77,441	19,114	~4	20	73178	23
Whitehead	On-target prediction	Conventional	182,134	18,166	~10	20	1000000067	19
Toronto KnockOut v3	On-target prediction and off-target prediction	Conventional	71,090	18,056	~4	20	90294	28

*Gene-targeting gRNAs only; excludes control gRNAs. This number differs from the gRNA totals in Fig. 1 because the latter are for the number of distinct 19-mer gRNAs.

**Figure 2:**
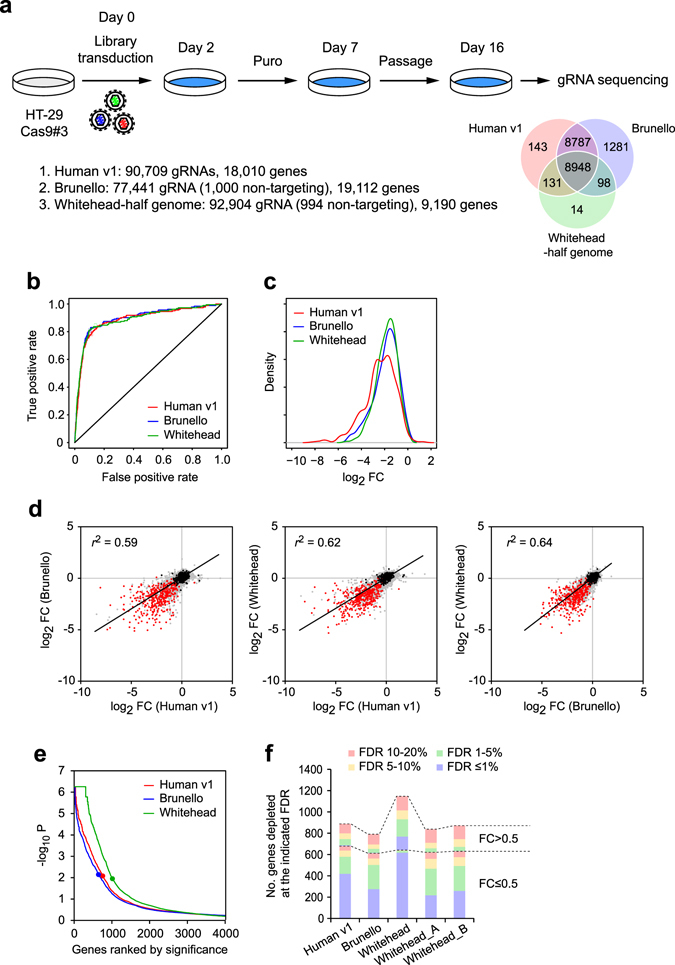
Screening performance of three second-generation CRISPR libraries. (**a**) Schematic showing the screening procedure and the libraries used in this study. (**b**) ROC curves showing the classification performance of the three CRISPR libraries on predefined set of essential and non-essential genes. (**c**) Density functions of gene-level fold change (FC) for 366 essential genes from each library, showing that the Human v1 library exhibits a left shift compared to the other two libraries. (**d**) Pairwise comparison of the gene-level log_2_ fold changes of the 8,948 genes analysed. Red and black dots represent the predefined essential and non-essential genes^31^, respectively. Correlation coefficient was shown for each comparison. Similar to c, the Human v1 library exhibits greater FC. (**e**) Plot showing gene rank by significance. (**f**) Number of depleted genes detected at the indicated FDR and FC by each library. The Whitehead library was also separated into two subsets denoted with suffix _A and _B.

To eliminate any bias that might be caused by differences in the lentiviral vector backbone used to generate each library, we re-constructed the complete Brunello^27^ and half of the Whitehead^36^ libraries on our lentiviral backbone (see Methods and Fig. 2a). Doing so also ensures that the three libraries had a similar level of complexity (77 k–92 k gRNAs) which allowed us to perform screens in an identical experimental setting. Screens were performed in the colon cancer cell line HT-29 Cas9#3 clone^37^ in technical triplicate and the gRNA abundance at 16 days post transduction was analysed (Fig. 2a). For meaningful comparisons we subsampled the read counts for 8,948 genes common to the three reconstructed libraries (Fig. 2a; read counts in Supplementary Table S1) in all our subsequent analyses. We obtained the false discovery rate (FDR) for these genes using the read counts from all three replicates using MAGeCK^39^, and separately calculated their respective gene-level fold change (FC).

The receiver operating characteristic (ROC) curves of all three libraries showed nearly identical performance in discriminating the essential from non-essential genes (Fig. 2b). We however found that the FCs in the Human v1 library exhibited a more significant reduction compared to in the other two libraries (Fig. 2c; Kolmogorov-Smirnov test, *p* = 2.8 × 10^−7^ and 5.3 × 10^−9^, against the Brunello and Whitehead libraries, respectively, and Fig. 2d). When the gene-level statistical results returned by MAGeCK were compared, the Whitehead library detected the largest number of significantly depleted genes (Fig. 2e). This is likely due to the fact that the Whitehead library comprises 10 gRNAs per gene, much higher than the Brunello (4 gRNAs per gene) and the Human v1 (5 gRNAs per gene) libraries.

We then integrated the FDR and FC values and counted the number of genes depleted at different FDR and FC cut-offs. The differences between the libraries immediately became more apparent (Fig. 2f). Nearly all genes with FC ≤ 0.5 in the Whitehead library were below 1% FDR, but the fraction of such genes are lower at 62% and 45% in the Human v1 and Brunello libraries, respectively. Although the Whitehead library detected the largest number of genes, approximately 45% of the genes detected below FDR 20% showed a smaller depletion (FC > 0.5). The fraction of such genes was smaller in the Human v1 (23.5%) and Brunello (22.6%) libraries. When the Whitehead library read count data were separated into two subsets (sets A and B, as described^36^), each of the 5 gRNAs-per-gene subsets detected comparable numbers of depleted genes and showed similar trends as the Human v1 and Brunello libraries (Fig. 2f). These results indicate that these second-generation CRISPR libraries show comparable screening performance and identify similar numbers of depleted genes when the number of gRNA per gene is similar. It is however worth noting that the fraction of genes with FDR ≤ 1% is biggest in the Human v1 library.

### Concordance of depleted genes detected and false negative rate estimation

**Figure 3:**
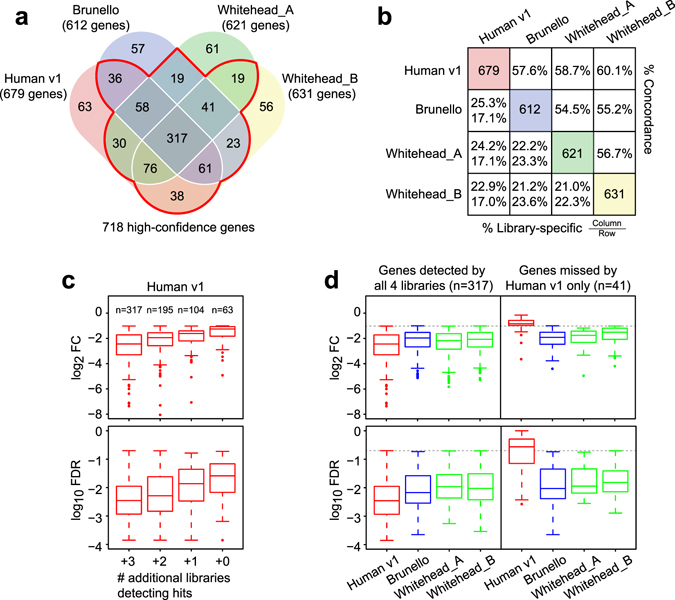
Properties of the depleted genes detected by three second-generation CRISPR libraries. (**a**) Venn diagram showing overlaps of genes detected at FDR ≤ 20% and FC ≤ 0.5. The Whitehead library was separated into two subsets denoted with suffix _A and _B. Genes detected by 2 or more libraries are considered as high-confidence genes. (**b**) Pairwise concordance between libraries of detected depleted genes. (**c**) Box plots showing the FC and FDR of genes detected by the Human v1 library. Genes were grouped by the number of additional libraries which detected those hits. See Supplementary Fig. S1a for the complete result. (**d**) Box plots showing the FC and FDR of genes detected by all 4 libraries and of genes missed only by the Human v1 library. See Supplementary Figure S1b for the complete result.

We next analysed the overlaps of genes that satisfied both cut-off criteria (FDR ≤ 20% and FC ≤ 0.5) in the Human v1, Brunello and the two Whitehead subset libraries (Fig. 3a). A total of 955 non-redundant genes were detected by these 4 libraries. Of these, 718 genes (75.1%) were detected by 2 or more libraries, which we dubbed as “high-confidence genes”. Within this confident set, 317 genes (44.2%) were detected by all 4 libraries. The concordance between any two given libraries was similar, at between 55% and 60%, but the Human v1 library consistently detected more genes than the other libraries (Fig. 3b). As demonstrated using the genes detected by the Human v1 library, the FCs and FDRs tend to be greater if the genes were detected by more libraries (Fig. 3c). In each library, library-specific hits accounted for approximately 10% of the total hits and they have a lower FC. These trends were consistent across all gRNA libraries tested (Supplementary Fig. S1a).

One important question that remains unanswered in CRISPR-KO screening is the false negative (FN) rate. As we do not know the phenotype of every gene KO in a given cell type, theoretically it is not possible to measure the true FN rate of CRISPR-KO screens. We sought to address this issue by utilising the high-confidence gene set we defined above to arrive at an estimate. Among the 718 high-confidence genes, the number of genes missed by each library was 102, 163, 158 and 143 for the Human v1, Brunello, Whitehead_A and Whitehead_B libraries, respectively (Fig. 3a), resulting in observed FN rates of between 14% and 23%. Among the FN genes of each library, the GO terms related to cell survival and proliferation were highly enriched (Supplementary Table S2), indicating that similar to commonly detected genes, these FN genes have fundamental cellular functions. As exemplified by the Human v1 library in Fig. 3d, FN genes from this library showed substantially lower FC and weaker significance but were detected by the other 3 libraries, albeit at a slightly weaker FC and FDR when compared to the 317 commonly-detected genes. A similar trend was observed in all other libraries for library-specific FN genes (Supplementary Fig. S1b). We then analysed the genes missed by 2 libraries and found a similar trend, although the FC of genes in this category was considerably lower than that of the commonly detected genes (Supplementary Fig. S1c). The gRNAs targeting the FN genes of a given library exhibited marked variation in their FC, many of which showed little to no effect on proliferation, whereas the gRNAs from the other libraries indeed produced phonotypic effects (Supplementary Fig. S2). We also calculated the FN rates by comparing the Human v1, Brunello and the Whitehead full-set and found FN rates of 9.9%, 18.5% and 9.3%, respectively (Supplementary Fig. S3). These results suggest that each library has a varying level of inherent false negatives and that false negatives are primarily caused by the selection and inclusion of less or non-effective gRNAs in the respective libraries.

### Optimal number of gRNAs per gene and number of replicates on screening performance

As reported previously^39^, the number of gRNAs per gene has a substantial impact on statistical results. We sought to revisit this question as the greater efficiency accorded by the second-generation libraries may affect the optimal parameters for CRISPR-KO screening. We cloned the 10 gRNAs-per-gene Whitehead sub-library used above into our lentiviral vector carrying the optimised scaffold^37, 38^ and performed the same screen (read counts in Supplementary Table S1). This library showed greater FC than the library using the conventional scaffold, indicating that the optimised scaffold can further improve gRNA efficacy even if the gRNAs were already selected based on on-target prediction scores (Supplementary Fig. S4).

**Figure 4:**
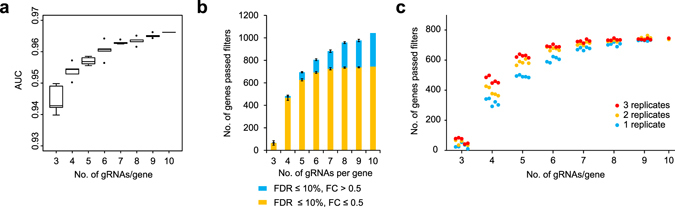
Optimal number of gRNAs per gene and number of replicates. (**a**) Box plots showing area under the curve (AUC) from ROC analysis using different gRNAs per gene. (**b**) The number of depleted genes detected at the indicated FDR and fold change (FC) cut-offs. (**c**) The number of depleted genes detected at FDR ≤ 10% and FC ≤ 0.5 from reduced numbers of replicates for each subset.

In order to assess the effect of the number of gRNAs per gene, we down-sampled this dataset and generate a series of read count subsets, ranging between 3 and 9 gRNAs per gene (5 subsets for each per-gene gRNA number) and performed MAGeCK and ROC curve analyses individually. As expected, the library with 10 gRNAs per gene exhibited the greatest area under the curve (AUC); however, even with fewer numbers of gRNAs, ROC curve analysis detected only marginal performance difference across the subsets (Fig. 4a). Consistent with our previous observation (Fig. 2f), greater difference became evident when we compared the numbers of depleted genes with FDR and FC cut-offs (Fig. 4b). The subsampled libraries with 3 gRNAs per gene detected few hits below 10% FDR, whereas the performance improved markedly in libraries with 4 gRNAs per gene. The total numbers of depleted genes detected below 10% FDR increased further as the number of gRNAs per gene increases up to 10 gRNAs per gene. However, when FC was also used as an additional criterion, the number of depleted genes with FDR ≤ 10% and FC ≤ 0.5 plateaued at around 6 gRNAs per gene, whereas the number of genes with FDR ≤ 10% and FC > 0.5 kept increasing. These results indicated that increasing the number of gRNAs per gene is beneficial to a certain point, but beyond this point the increase in significance only accounts for genes with a minor effect (as indicated by their low FC) and thus are not crucial to the phenotype in question.

We next analysed the effect of the number of replicates on the screening outcome. For each down-sampled dataset, we performed statistical analysis with reducing number of replicates from 3 to 2 to 1 and counted the genes with FDR ≤ 0.1 and FC ≤ 0.5 (Fig. 4c). This analysis revealed that conducting a screen in more replicates improved sensitivity and detected a greater numbers of genes, particularly when using libraries with 4 or 5 gRNAs per gene. However, the difference between 2 and 3 replicates became negligible in libraries that contain 6 or more gRNAs per gene. Moreover, when using libraries with 9 gRNAs per gene, screens with a single replicate were as robust as those with 2 or more replicates.

**Figure 5:**
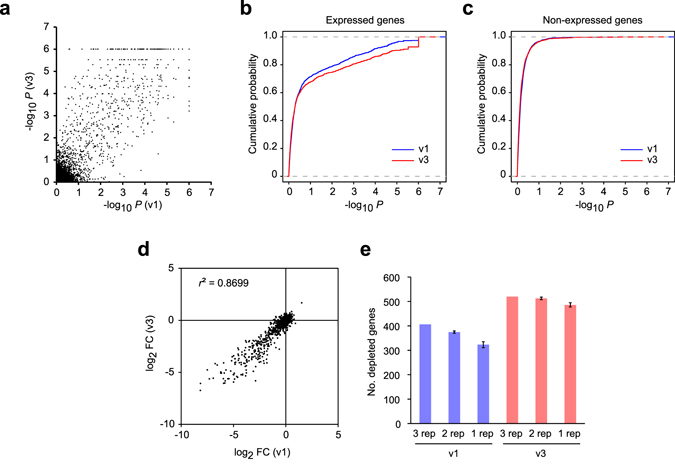
Screening performance of the 5-gRNAs-per-gene Human v1 and 6-gRNAs-per-gene Human v3 libraries and the effect of replicates. (**a**) Scatter plot comparing gene-level significance between the Human v1 and v3 libraries. (**b**,**c**) Cumulative distribution function of gene-level significance of expressed (**b**) and non-expressed (**c**) genes. Non-expressed genes are defined as having FPKM values of less than 0.5 and expressed genes as having FPKM values of 0.5 and above. (**d**) Scatter plot comparing the fold change between the Human v1 and v3 libraries. (**e**) The number of depleted genes detected by the Human v1 and v3 libraries with different number of replicates.

Taken together, we concluded that screens using a second-generation library with 6 gRNAs per gene in duplicate would provide the most suitable and practical trade-off. To evaluate the sensitivity of libraries with 6 gRNAs per gene, we re-designed our library and generated new Human v3 library, yielding a total of 114,749 gRNAs targeting 18,740 human genes (Supplementary Table S3). To test this new v3 library and to evaluate the screening parameters we had determined, we randomly selected 5,000 genes as a test set and cloned an oligo pool into a modified version of our lentiviral vector (Supplementary Fig. S5) and similarly performed a screen in HT-29 cells (read counts in Supplementary Table S4). The new lentiviral gRNA vector incorporated a library-specific barcode, which can be used for a reverse primer-annealing site, thus preventing gRNA amplification from existing lentiviral gRNA vectors due to cross-contamination. The vast majority of depleted genes with the new v3 library showed improved significance (Fig. 5a). Further corroboration using RNA-seq data^37^ also showed the v3 library as producing higher significance in the expressed genes but not in the non-expressed genes (Fig. 5b,c). As the principle of gRNA selection and the gRNA scaffold used remained unchanged, there was no significant difference in the FC between the v1 and v3 libraries (Fig. 5d; Kolmogorov-Smirnov test, *p* = 0.44). We next conducted statistical analysis with various numbers of replicates and compared the number of depleted genes (Fig. 5e). Consistent with the observation in Fig. 4c, there was almost no difference in the number of depleted genes between the 3- and 2-replicate screens with the v3 library. Even with a single replicate, the reduction in detecting depleted genes was marginal and the new library could thus detect more genes than the three-replicate screen with the v1 library.

## Discussion

Prior to the advent of CRISPR-Cas9-based genome editing technology, genome-wide recessive screening had been typically performed using the RNA interference technology^40^. However, the intrinsic nature of RNAi, namely incomplete silencing and frequent off-target effects, resulted in low detection sensitivity and inconsistent results across laboratories. In contrast, the CRISPR-Cas9 system directly modifies genomic DNA and generates inactivating mutations, thereby exhibiting greater gene perturbation efficiency and stronger phenotypic outcomes. This was well exemplified by a recent study on CRISPR-KO screens for host factors required for HIV-1 infection^41^. Nonetheless, the CRISPR-Cas9 system has several pitfalls. As a result of nucleic acid-based recognition of targets, the CRISPR-Cas9 system could exhibit off-target effects through imperfect hybridisation between a gRNA and target DNA^23, 42^. A few computational prediction methods have been developed^23, 27^, but predicting which potential off-target sites are actually cleaved is still difficult. Selecting gRNAs with the fewest predicted off-target sites would still be the most practical approach.

Another major pitfall of CRISPR-KO screening, especially screens in cancer cell lines, is the false positives caused by copy number aberration^43, 44^. As CRISPR-KO screening relies on double-stranded DNA break (DSB) generated by wildtype Cas9 protein to inactivate gene function, the higher number of DSBs in copy-number-amplified regions generate higher genotoxicity than those in normal copy number regions, causing cell proliferation delay or deletion of cancer driver genes in such regions. As a result, genes without any proliferative impact may be falsely identified as a screen hit. Gene copy number data should ideally be incorporated when interpreting screening results from cancer cell lines to minimise false-positive calls.

CRISPR-KO screening using the first-generation libraries did not robustly detect genes in negative selection. In order to improve screening performance, two different approaches have been taken. One approach is to optimise the gRNA scaffold sequences. Several studies have been conducted thus far and resulted in a similar structure, which showed more robust phenotypic outcomes^38, 45, 46^. Another approach is to computationally predict on-target efficiency using nucleotide biases on gRNA KO efficiency. These improvements have been adopted when generating the second-generation CRISPR-KO libraries.

The degree of differences between the four second-generation CRISPR libraries may not be immediately apparent with a quick glance at Table 1. However, the overlapping patterns of the human genes targeted and the constituent gRNAs in Fig. 1 revealed that only a measly 0.3% of the 350,860 unique gRNAs are shared by all four libraries, and only about 16% are present in more than one library, despite having 17,070 genes (87.1% of the total) targeted by all four libraries. Even the three libraries that were designed using on-target prediction algorithms (Table 1) shared only 4,002 gRNAs, a mere 1.42% of the three-library 281,418 gRNA pool. This great diversity of gRNAs is a testament to the huge effect that the gRNA design principles have on the composition of CRISPR libraries.

Despite the huge differences in the composition of the gRNAs and other parameters (Table 1), the estimated false negative rates of the four libraries (Human v1, Brunello and the two Whitehead subsets) turned out to be remarkably similar at between 14% and 23% (at FDR ≤ 20% and FC ≤ 0.5). This indicates that each of the libraries has their fair share of gRNAs which are not active in producing gene knockouts. This also suggests that false negatives are difficult to avoid simply via currently-available computational methods of gRNA selection, as the elaborate on-target prediction algorithms did not show significant advantage over the gRNA design principles we used for the Human v1 library. Further work is required to understand factors that influence gRNA efficacy. It would also be worth considering the construction of new CRISPR libraries via selecting and incorporating validated active gRNAs when more CRISPR-KO datasets using different libraries become available.

Our *in silico* subsampling analyses from the 10-gRNA-per-gene Whitehead half library (Fig. 4) identified a substantial gRNA number-dependent effect on statistical results returned by MAGeCK. It is evident from our results that a higher number of gRNAs per gene allows for a more robust statistical analysis but can become over-sensitive with the tendency of calling genes with a relatively minor phenotypic effect (i.e. lower fold change values) as significant hits. From the perspective of practicality, a higher number of gRNAs per gene would cause the following two disadvantages. Firstly, increasing the numbers of gRNAs per gene invariably results in a larger library size. As a consequence, ensuring that the library complexity is maintained throughout the screening process would become increasingly challenging, and the loss of library complexity could lead to poor screening outcome. The second shortcoming of using a higher number of gRNA per gene means over-detecting hits, resulting in unnecessary downstream validation work. Therefore, it is important to identify a practical trade off that reports biologically meaningful outcome with libraries small enough to perform a proper screen. For second-generation CRISPR-KO libraries, we have showed that the use of a library containing 6 gRNAs per gene in duplicated screens is likely to be the optimal design parameter for genome-wide CRISPR-KO screens. In the validation screen of our new Human v3 library, we have detected with just a single replicate significantly more depleted genes than duplicated screens using our v1 library (Fig. 5e). This translates to a reduction of 60% in required reagents and laboratory work such as tissue culture and sequencing. For data analysis, several statistical packages have been developed and more are likely to be developed^47–50^. To narrow down a large number of primary hits to high confidence hits, additional statistical analyses using these newly developed tools would be of great utility.

CRISPR-KO screens in cancer cell lines have been successfully conducted and have identified novel therapeutic targets^34, 36, 37, 51, 52^. There will be, however, more demands in screening cell types less amenable to scaling up, such as organoids and primary cells^53^. Our newly defined optimal screening parameters would be helpful to other researchers in the field who are considering conducting such screens with genome-wide libraries or constructing bespoke gRNA libraries.

## Methods

### Plasmid construction

pKLV3-U6gRNA5(BbsI)-PGKpuroBFP-W-L1 was constructed by cloning a gBlock fragment containing the gRNA expression cassette and a bar code into the MluI-BamHI site of pKLV2-U6gRNA5(BbsI)-PGKpuroBFP-W^37^ using a Gibson assembly kit (NEB).

### CRISPR libraries

The Brunello library was purchased from Addgene (catalog #73178). The gRNA expression cassette of the Brunello library was PCR-amplified with primers (5′-TAGTACCGGGCCCTACGCGTGAGGGCCTATTTCCCATG-3′ and 5′-CTACCCGGTAGAATTGGATCCAAAAAAAGCACCGACTCG-3′). The resulting PCR product was purified with a gel purification kit (Qiagen) and cloned into the MluI-BamHI site of pKLV2-U6gRNA5(BbsI)-PGKpuroBFP-W using a Gibson assembly kit (NEB). Five assembly reactions were performed with 100 ng of the vector and 9.1 ng of the insert fragment per reaction at 50 °C for 30 min. The reactions were then combined, purified with a MinElute PCR purification kit (Qiagen) and eluted into 10 µl water. Assembled DNA was electroporated into DH10B electro-competent cells (NEB) with 1 µl per reaction. All 10 reactions were combined, incubated at 37 °C for 1 hr and directly inoculated into 500 ml 2xTY medium containing 50 µg ml^−1^ ampicillin. Plasmid DNA was purified from overnight culture using a Plasmid Maxi kit (Qiagen).

For the Whitehead library (Addgene catalog #1000000067), gRNA sequences for randomly selected 9,190 genes were obtained from the published gRNA list. The sequences were appended as follows: 5′-GCAGATGGCTCTTTGTCCTAGACATCGAAGACAACACCGN_20_GTTTTAGTCTTCTCGTCGC-3′. Pooled oligos was purchased from CustomArray and cloned into pKLV2-U6gRNA5(BbsI)-PGKpuroBFP-W as described previously^21^.

The v3 library was designed essentially as described previously^37^ with a minor modification. We added another gRNA selection filter of GC% being 40–80% and selected 6 gRNAs per consensus coding sequence (CCDS). An oligo pool containing 30,000 gRNAs targeting 5,000 randomly selected genes was purchased from Twist Bioscience and cloned into pKLV3-U6gRNA5(BbsI)-PGKpuroBFP-W-L1 as described previously^21^.

### Virus production, transduction, screening, gRNA amplification and sequencing

These were performed as described previously^21, 37^. To amplify gRNA fragments from the v3 library, 5′-ACACTCTTTCCCTACACGACGCTCTTCCGATCTCTTGTGGAAAGGACGAAACA-3′ and 5′-TCGGCATTCCTGCTGAACCGCTCTTCCGATCTTACCCAGACTGCTCATCGTC-3′ were used.

### Processing of gRNA count tables

All FDR values were calculated using MAGeCK version 0.5.3^39^.

Prior to the calculation of fold change (FC) values, all gRNA count values were first normalised against the total of the baseline plasmid counts to account for the sequencing yield variability between replicates, then incremented by 1 to avoid division-by-zero errors. The incremented gRNA counts for the replicates were then totalled and averaged, before division by the gRNA baseline plasmid read count to arrive at the gRNA-level fold change. Gene-level FC values were calculated as the average of the fold change values of all constituent gRNAs for each gene.

### ROC analysis

ROC analysis were done in R, using predefined lists^31^ of non-essential genes (n = 927) and essential genes (n = 684).

### Data Availability

All data generated or analysed during this study are included in this published article and its Supplementary Information files.

## Electronic supplementary material

Supplementary Information

Supplementary Table S1

Supplementary Table S3

Supplementary Table S4
