# Author Correction: Optimised metrics for CRISPR-KO screens with second-generation gRNA libraries

**DOI:** 10.1038/s41598-018-24092-w

**Published:** 2018-04-12

**Authors:** Swee Hoe Ong, Yilong Li, Hiroko Koike-Yusa, Kosuke Yusa

**Affiliations:** Wellcome Trust Sanger Institute, Hinxton, Cambridge CB10 1SA UK

Correction to: *Scientific Reports* 10.1038/s41598-017-07827-z, published online 07 August 2017

This Article contains typographical errors in Table 1.

For the Human v1, Brunello, Whitehead and Toronto KnockOut v3 libraries, the references should read ‘37’, ‘27’, ‘20’ and ‘31’ respectively.

