# Supplementary material for: Optimised metrics for CRISPR-KO screens with second-generation gRNA libraries
Source: Sci Rep. 2017 Aug 7;7:7384. doi: 10.1038/s41598-017-07827-z (PMC5547152; doi:10.1038/s41598-017-07827-z)
Supplement: Supplementary file 1 — Supplementary Information [file 41598_2017_7827_MOESM1_ESM.pdf]

Supplementary Information

Optimised metrics for CRISPR-KO screens with second-generation gRNA libraries

Swee How Ong, Yilong Li, Hiroko Koike-Yusa and Kosuke Yusa

Supplementary Information

Supplementary Figure S1. Fold change (FC) and false discovery rate (FDR) for genes in different categories. **a,** In each library, genes were grouped based on the number of additional libraries detecting the genes at FDR ≤ 20% and FC ≤ 0.5. Genes detected by multiple libraries show greater FC and FDR than those detected by fewer libraries. **b**, In each library, library-specific FN genes show lower FC and weaker significance than the other libraries. FC and FDR from the other libraries detecting FN gene in a given library are slightly weaker but comparable to those of the genes commonly detected by all 4 libraries. **c**, FN genes by two libraries also show a similar pattern; however, in this group, detecting libraries show significant but substantially lower FC and weaker significance, compared to commonly detected genes.

Supplementary Figure S2. FC of individual gRNAs from each replicate for selected library-specific FN genes for each library. gRNAs targeting FN genes tend to show greater variation or no activities, resulting in weaker significance and lower FC.

Supplementary Figure S3. Concordance and false negatives analysed with the results from Human v1, Brunello and Whitehead full-set. **a**, A total of 862 non-redundant genes were detected, of which 659 genes (76.5%) were identified by 2 or more libraries and were considered as "high-confidence genes" in this particular analysis. Approximately 60% of the confident set was detected by all 3 libraries. Using this confident set, we calculated the FN rate for each library and found it to be 9.9, 18.5 and 9.3% for the Human v1, Brunello and Whitehead libraries, respectively. **b**, In pair-wise comparisons, the Human v1 and Whitehead libraries showed the highest concordance (67.7 %), whereas the Brunello library showed the lowest concordance with the other libraries (55-58 %).

Supplementary Figure S4. A comparison of gene-level FC between the conventional and improved gRNA scaffold. The same oligo pool (Whitehead-half genome library) was used to construct these libraries.

Supplementary Figure S5. A modified version of the lentiviral gRNA expression vector used for constructing the Human v3 library. a, A barcode sequence was inserted between the gRNA scaffold and the Pgk promoter and used for the reverse-primer annealing site. b, A new primer pair (U1 and U3) amplifies PCR products from the new vector but not from the previous vector. This allows us to specifically amplify gRNAs from library constructs even if cross-contamination of gRNAs from the previous vector occurs.

Supplementary Table S1. Read counts for the Human v1, Brunello and Whitehead libraries (with both the conventional and improved scaffold) for the overlapped 8,948 genes.

Supplementary Table S2. GO term enrichment in the false negative genes from each library.

Supplementary Table S3. List of gRNAs in the Human v3 library.

Supplementary Table S4. Read counts for the 5,000 genes used for the Human v1-v3 libraries comparison.

Supplementary Figure S1

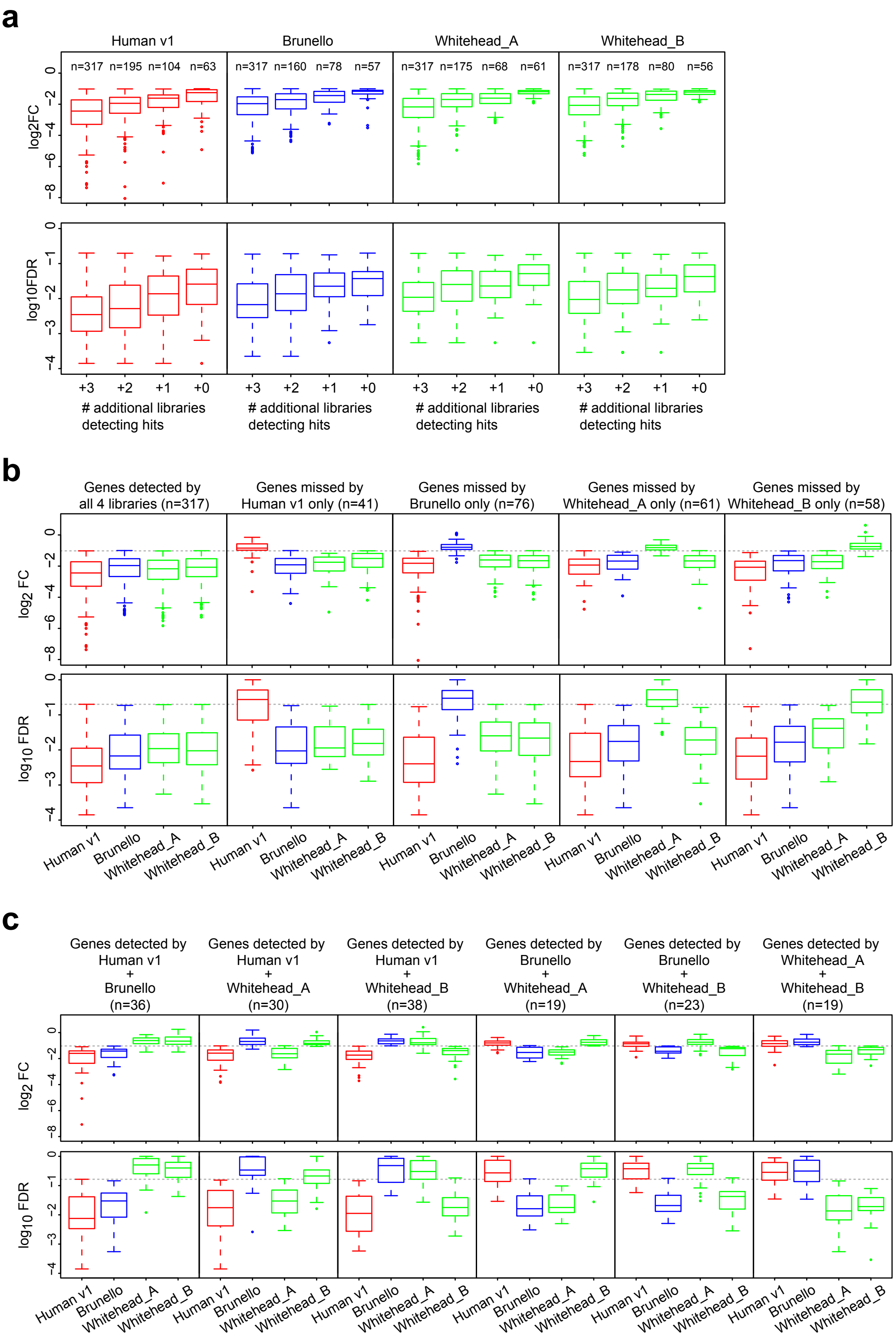

Supplementary Figure S2

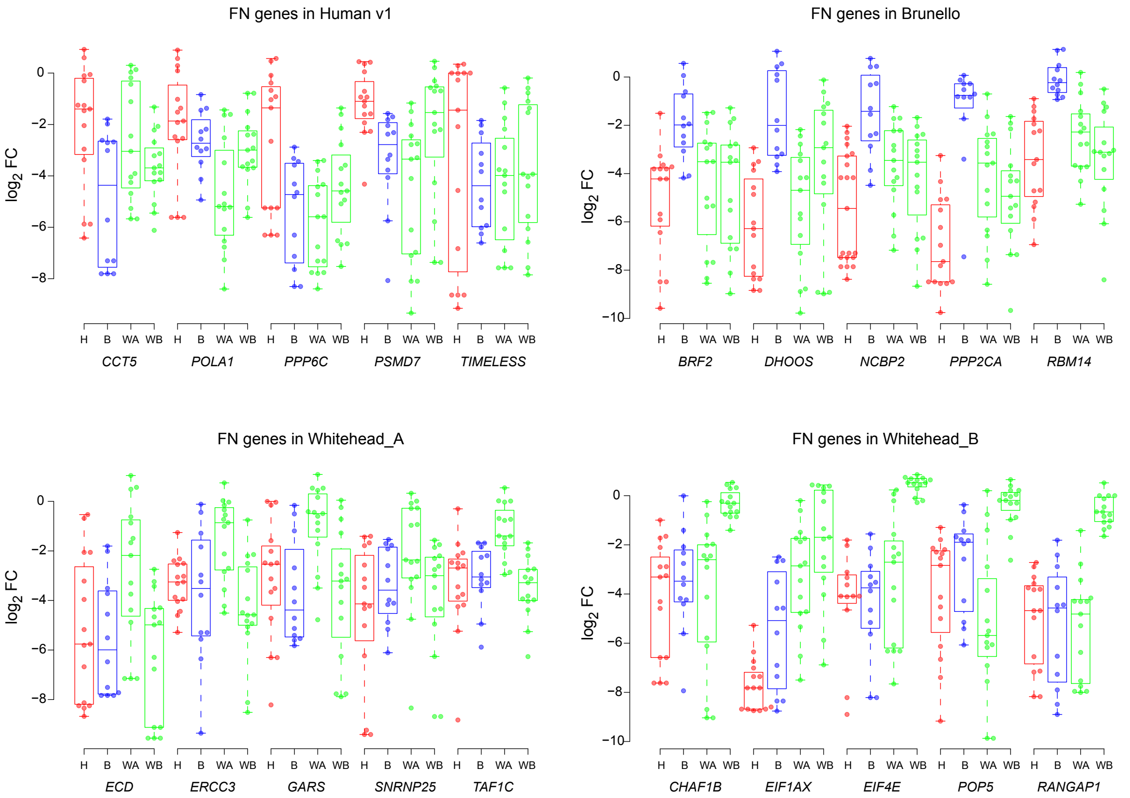

Supplementary Figure S3

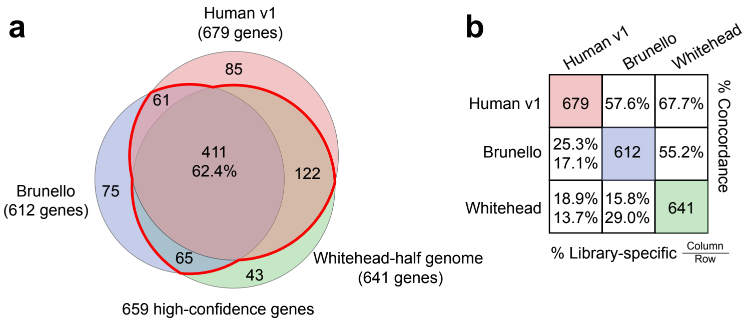

Supplementary Figure S4

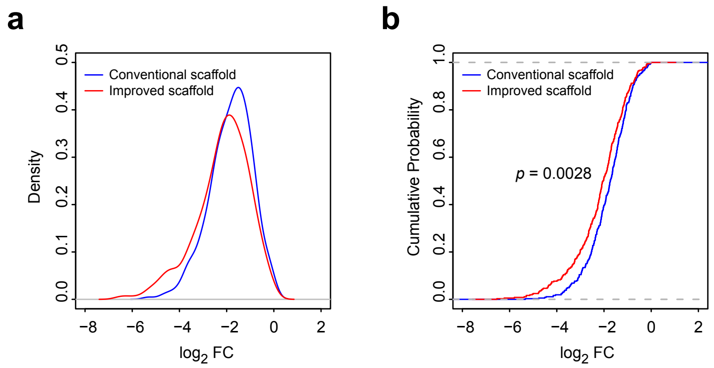

Supplementary Figure S5

a

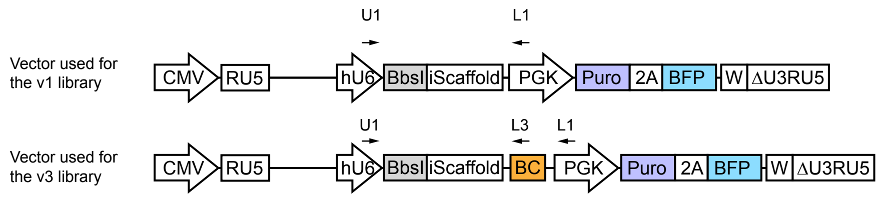

b

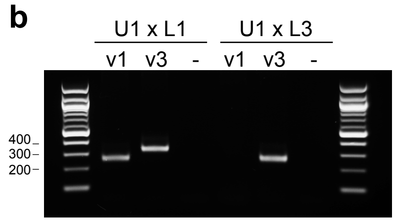

Supplementary Table S2. Enrichment of GO biological processes in the false negative genes among the defined high-confidence gene sets.

Gene Set Name	Human v1		Brunello		Whitehead_A		Whitehead_B	
	# Genes in Overlap (k)	FDR	# Genes in Overlap (k)	FDR	# Genes in Overlap (k)	FDR	# Genes in Overlap (k)	FDR
GO_CELL_CYCLE	12	8.52E-07					16	3.02E-09
GO_CELL_CYCLE_PHASE_TRANSITION	7	1.66E-06						
GO_CELL_CYCLE_PROCESS	11	1.28E-06					13	2.13E-07
GO_CELLULAR_MACROMOLECULAR_COMPLEX_ASSEMBLY					12	1.04E-07	13	3.21E-09
GO_MACROMOLECULAR_COMPLEX_ASSEMBLY					18	1.43E-10	18	1.03E-10
GO_MACROMOLECULE_CATABOLIC_PROCESS	11	5.92E-07						
GO_MATURATION_OF_SSU_RRNA			5	2.89E-06				
GO_MITOTIC_CELL_CYCLE	10	8.52E-07					11	7.35E-07
GO_MRNA_METABOLIC_PROCESS	10	2.99E-07	15	4.32E-11	17	5.39E-15	11	9.83E-08
GO_MRNA_PROCESSING			11	4.01E-08	13	2.01E-11	10	7.56E-08
GO_NCRNA_METABOLIC_PROCESS			17	1.31E-14				
GO_NCRNA_PROCESSING			12	7.93E-10				
GO_NUCLEAR_TRANSCRIBED_MRNA_CATABOLIC_PROCESS_NONSENSE_MEDIATED_DECAY	6	8.52E-07						
GO_ORGANONITROGEN_COMPOUND_BIOSYNTHETIC_PROCESS					13	3.14E-07		
GO_PROTEIN_COMPLEX_SUBUNIT_ORGANIZATION					15	3.77E-07		
GO_RIBONUCLEOPROTEIN_COMPLEX_BIOGENESIS	8	2.48E-06	12	2.61E-09	10	1.67E-07		
GO_RIBOSOME_BIOGENESIS	7	4.88E-06	11	1.42E-09				
GO_RNA_PROCESSING	11	2.99E-07	22	9.68E-18	19	3.27E-15	14	1.95E-09
GO_RNA_SPLICING			9	2.89E-06	10	4.59E-08	9	2.85E-07
GO_RNA_SPLICING_VIA_TRANSESTERIFICATION_REACTIONS					9	5.83E-08	9	3.09E-08
GO_RRNA_METABOLIC_PROCESS			11	2.75E-10				
